# Human testicular organoid system as a novel tool to study Zika virus pathogenesis

**DOI:** 10.1038/s41426-018-0080-7

**Published:** 2018-05-09

**Authors:** Daniel P. Strange, Nima Pourhabibi Zarandi, Goral Trivedi, Anthony Atala, Colin E. Bishop, Hooman Sadri-Ardekani, Saguna Verma

**Affiliations:** 1 Department of Tropical Medicine, Medical Microbiology, and Pharmacology, John A. Burns School of Medicine, University of Hawaii at Manoa, Honolulu, HI 96813 USA; 2 Wake Forest Institute for Regenerative Medicine (WFIRM), Winston-Salem, NC USA; 3 Department of Urology, Wake Forest School of Medicine, Winston-Salem, NC 27101 USA

The 2015–2016 Zika virus (ZIKV) epidemic, resulting in >1.5 million cases in the Americas, emerged as a global threat to public health due to the unique findings of sexual and in utero transmission, which have not been reported for other flaviviruses^1^. In ZIKV endemic regions, the role of sexual transmission in disease spread may be difficult to predict; however, a recent study demonstrated that 56% of ZIKV serum-positive males were also semen-positive for virus for up to 108 days after infection^2^. This suggests a higher contribution in disease spread and a longer infectious phase as compared to other mosquito-borne flaviviruses. Further, Joguet et al.^3^ reported significantly higher seminal viral loads than in other bodily fluids of ZIKV infected men. This was accompanied by testosterone decline and significant decrease in sperm count early after symptoms onset, indicating ZIKV infection may also affect fertility^3^. The mammalian testis is divided into two compartments: seminiferous tubules composed of Sertoli cells (SC), peritubular cells, and various generations of germ cells (spermatogonial stem cells (SSC), meiotic spermatocytes, and spermatozoa); and interstitial space (between seminiferous tubules) consisting of blood vessels, tissue-resident immune cells, and testosterone-producing Leydig cells (LC)^4^. The testicular immune environment is tightly governed by communication between resident cell types, notably SC and LC, through production of transforming growth factor beta (TGF-β), testosterone, activin, and inhibin complexes, as well as pro-inflammatory cytokines and type I Interferons^5^.

Little is known of the cell types that support ZIKV persistence within the human testes. We recently demonstrated that primary human SC are highly susceptible to ZIKV infection compared to dengue virus, and are capable of robust antiviral and inflammatory responses^6^. Using an in vitro Sertoli cell barrier (SCB) model we further demonstrated that ZIKV could cross the barrier more efficiently compared to dengue virus without altering barrier permeability^6^. However, the limitation of monolayer two-dimensional (2D) culture systems is that they do not allow assessment of multiple testicular cell interactions and the effect of ZIKV on the fate of germ cells. Thus, novel tools that mimic functionality of native tissue to study complex consequences of ZIKV infection are greatly needed. Previous studies have used three-dimensional (3D) mouse testicular tissue cultured on soft-agar, but a human 3D culture model is considered optimal to mimic human testes function^7^. A restricted number of research groups, including ours have reported and characterized testicular organoids, as organ-like structures that relatively model testicular histology and physiology by way of reorganization of dissociated testicular cells in vitro^8–10^. Our recently developed human testicular organoid (HTO) culture system is composed of SSC, SC, LC, and peritubular cells that maintain >85% viability throughout long-term culture of 23 days^10^. These HTO also produce testosterone continuously and support limited germ cell differentiation, and thus partially recapitulate the composition, diversity, and function of human testes^10^.

**Fig. 1:**
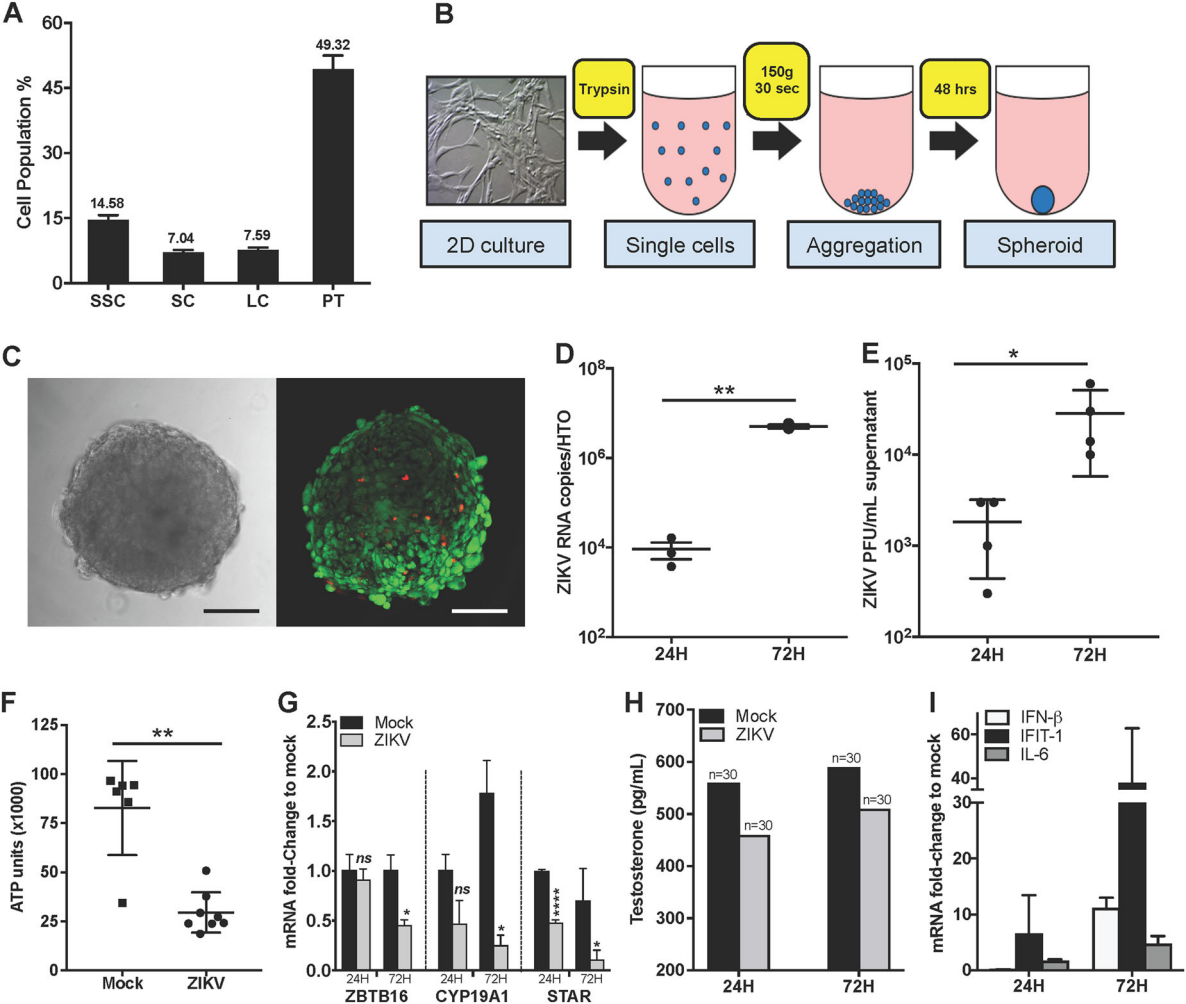
ZIKV productively infects human testicular organoids. **a** Percentage of cell types in 2D propagated primary human testicular cell cultures, using Digital PCR detecting single cell per well (*n* = 3, error bars represent 95% confidence interval). D-PCR markers: ZBTB16 (spermatogonia cells), SOX9 (Sertoli cells), STAR (Leydig cells) and ACTA2 (Peritubular cells). **b** Generation of 3D HTO by centrifugation (150 g for 30 s) of SSC, SC, LC, and peritubular cells in ultra-low attachment 96-well plate resulting in spheroid formation in 48 h. **c** Morphology and viability evaluation of HTO. (Left) Bright field imaging. (Right) LIVE/DEAD Confocal 3D Z-Stack projection of a representative organoid after freezing and post-thaw recovery in culture (red fluorescent depicts dead cells and green depicts live cells). Scale bar 100 µm. ZIKV titers measured from infected HTO cultures by qRT-PCR (**d**) and plaque assay (**e**) demonstrated significant increase at 72 h (*n* = 10 HTO/data point). **f** Evaluation of ATP production (relative luminometer units, RVU) per organoid using CellTiter-Glo® 3D Cell viability assay depicted significant decrease in viability 72 h after infection (*n* = 6–8 HTO/time point). **g** qRT-PCR for markers for spermatogonial cells (ZTBT16), SC and LC (CYP19A1), and LC (STAR) at 24 and 72 h after infection. **h** Testosterone concentrations (pg/mL) were assessed by automated competitive binding immunoenzymatic assay (UniCel DXI 800, Beckman Coulter, Inc.). The data represent mean values of media pooled from 30 HTO per condition/time point. **i** Peak virus titers correlated with increased expression of antiviral genes IFN-β, IFIT1, and IL-6 (data represent mean of triplicate samples and each RNA sample was from 10 pooled HTO). *p ≤ 0.05; **p ≤ 0.01; ****p ≤ 0.0001

To determine whether HTO developed from primary cultured testicular cells can be utilized to study ZIKV infection, we first assessed their susceptibility to ZIKV. The HTO were formed using 2D propagated primary LC, SC, peritubular cells, and spermatogonia (Fig. 1a) from normal adult testicular tissue, by self-aggregation for 48 h in enriched medium containing testis extracellular matrix (ECM) extracted in our laboratory inside ultra-low attachment surface 96-well plates (10,000 cells/well, Fig. 1b) and then frozen slowly in DMSO based cryomedia as described previously^10^. Representative bright field and LIVE/DEAD imaging by confocal composite Z-stack projections of HTO in culture demonstrates that they maintain structure and viability in vitro post-thaw (Fig. 1c). For ZIKV infection, post-thawed HTO were cultured in ultra-low attachment round bottom 96-well plates in 200 µL of Complete StemPro-34 media (containing growth factors and ECM)^10^. After 5 days of recovery and reaching viability in range of fresh HTO, they were washed three times with StemPro-34 media without ECM and then infected with 10^5^ PFU of ZIKV (PRVABC59) in 100 µL for 1 h at 37 °C. This infectious dose was selected to mimic virus levels observed in blood during peak viremia^3^ and to ensure access of virus to cells at the inner core of HTO. Post-infection, the HTO were washed three times to remove the unbound virus and replenished with 200 µL fresh Complete StemPro-34 media. At 24 and 72 h after infection, media were collected and HTO (10 HTO pooled per sample) were washed three times with PBS and used for RNA extraction. Low copies of ZIKV RNA were detected at 24 h and increased significantly by >2 logs at 72 h (Fig. 1d). A similar trend was observed by plaque assay (Fig. 1e), collectively indicating that HTO can support productive ZIKV infection. We next evaluated the effect of ZIKV on overall HTO survival by measuring ATP production as a measure of cell viability. We observed a dramatic decrease in ATP at 72 h (three times lower as compared to controls, Fig. 1f) that inversely correlated with peak virus copy numbers. To investigate the effect of ZIKV on each cell type, we measured gene expression of markers for SC and LC (CYP19A1), LC (STAR), and undifferentiated spermatogonia including SSC (ZBTB16)^10^. As depicted in Fig. 1g, qRT-PCR for undifferentiated spermatogonial cell (including SSC) marker ZTBT16 demonstrated a 50% reduction in expression in infected HTO at 72 h. Similarly, expression of somatic cell markers CYP19A1 and STAR was also reduced significantly at 72 h after infection (Fig. 1g). Furthermore, testosterone production by infected HTO declined as compared to controls at both time points (Fig. 1h).

This preliminary study provides first evidence that ZIKV can efficiently infect testicular organoids. The decrease in the overall viability (Fig. 1f) and reduced expression of spermatogonial and somatic cell markers in infected HTO (Fig. 1g) suggests that multiple cell types are susceptible to cell death following ZIKV infection, most likely via apoptosis. Although mouse studies have characterized cellular targets of ZIKV and immune response in the testes^11–14^, deficiency in IFN response limits their human predictive capability. A clear strength of this HTO system would be to utilize it to delineate unique immune responses of different testicular cells to ZIKV. Our results as shown in Fig. 1i demonstrate induction of genes associated with antiviral defense such as IFN-β, IFIT-1 (an important IFN stimulated gene), and IL-6. Collectively, these preliminary results support the hypothesis that HTO can be used as a much-needed tool to understand the immune mechanisms of human testicular persistence of ZIKV. Since SSC cannot be cultured alone and require somatic cells-SSC niche, HTO also provide a valuable resource to characterize direct effects of ZIKV on early stages of germ cell differentiation.

Major gaps in our understanding of ZIKV infection in the human testes limit our efforts to ultimately develop therapeutic strategies to specifically clear testicular infection. This HTO model has so far been used to study gonadotoxicity^11^ but, in spite of the apparent limitation of only partial maturation of spermatids and lack of blood-testes barrier, has several applications even beyond modeling ZIKV infection. This study opens up opportunities to dissect infection kinetics and immune mechanisms of ZIKV persistence in different testicular cell types, provides a tool for investigating other potential testes-tropic viruses such as Ebola^15^, and more importantly reduces dependency on animal models. Future research using this HTO model to delineate unique cell type-specific response associated with long-term ZIKV replication in the testes will provide a framework for future basic and translational research, including evaluation of efficacy and toxicity of anti-ZIKV drugs for clearing testicular infections.
